# Evaluation of Suitable Polymeric Matrix/Carriers during Loading of Poorly Water Soluble Drugs onto Mesoporous Silica: Physical Stability and In Vitro Supersaturation

**DOI:** 10.3390/polym16060802

**Published:** 2024-03-13

**Authors:** Afroditi Kapourani, Konstantinos Katopodis, Vasiliki Valkanioti, Melina Chatzitheodoridou, Christos Cholevas, Panagiotis Barmpalexis

**Affiliations:** 1 Laboratory of Pharmaceutical Technology, Division of Pharmaceutical Technology, School of Pharmacy, Faculty of Health Sciences, Aristotle University of Thessaloniki, 54124 Thessaloniki, Greece; akapourag@pharm.auth.gr (A.K.); kkatopod@pharm.auth.gr (K.K.); vvasilik@pharm.auth.gr (V.V.); ccholevas@pharm.auth.gr (C.C.); 2 Natural Products Research Center of Excellence-AUTH (NatPro-AUTH), Center for Interdisciplinary Research and Innovation (CIRI-AUTH), 57001 Thessaloniki, Greece

**Keywords:** amorphous solid dispersions, mesoporous carriers, Syloid 244 FP, sustained supersaturation, physical stability

## Abstract

The application of mesoporous carriers in formulations of amorphous solid dispersions (ASDs) has been suggested to enhance the stability of amorphous drugs. However, mesoporous carriers do not demonstrate satisfactory inhibitory effects on the precipitation of active pharmaceutical ingredients (APIs), and the inclusion of an appropriate polymer within ASDs becomes imperative to maintaining drug supersaturation. The aim of this study was to evaluate ternary olanzapine (OLN) ASDs with Syloid 244FP and to find an appropriate polymeric carrier. The polymer’s selection criteria were based on the physical stability of the ASDs and the release rate of the drug from the systems. The polymers investigated were hydroxypropylmethyl cellulose (HPMC) and copovidone (coPVP). The formation of ASDs was achievable in all investigated cases, as demonstrated by the complete lack of crystallinity confirmed through both powder X-ray diffraction (pXRD) analysis and differential scanning calorimetry (DSC) for all developed formulations. The solvent shift method was employed to evaluate the ability of the studied carriers to inhibit the precipitation of supersaturated OLN. coPVP emerged as a more suitable precipitation inhibitor compared with HPMC and Syloid 244 FP. Subsequently, in vitro dissolution studies under non-sink conditions revealed a higher degree of supersaturation in ternary systems where coPVP was used as a polymeric carrier, as these systems exhibited, under the examined conditions, up to a 2-fold increase in the released OLN compared with the pure crystalline drug. Moreover, stability studies conducted utilizing pXRD demonstrated that ternary formulations incorporating coPVP and Syloid 244 FP maintained stability for an extended period of 8 months. In contrast, binary systems exhibited a comparatively shorter stability duration, indicating the synergistic effect of coPVP and Syloid 244 FP on the physical stability of the amorphous API. Attenuated total reflectance–Fourier transform infrared (ATR-FTIR) studies showed that the development of stronger molecular interactions can be provided as an explanation for this synergistic effect, as the formation of robust H-bonds may be considered responsible for inhibiting the precipitation of the supersaturated API. Therefore, the incorporation of coPVP into OLN ASDs with Syloid 244 FP is considered a highly promising technique for increasing the degree of OLN supersaturation in in vitro dissolution studies and improving the stability of systems.

## 1. Introduction

A pivotal challenge faced by the pharmaceutical industry is the pursuit of cost-effective strategies to improve drugs’ aqueous solubility [1,2]. This challenge is underscored by the fact that 40% of marketed active pharmaceutical ingredients (APIs) and more than 90% of new chemical entities belong to biopharmaceutics classification system (BCS) class II or IV [3,4]. This classification implies that these pharmaceutical compounds encounter solubility problems in gastrointestinal (GI) fluids, and hence, limited bioavailability when administrated orally [5,6]. As a result, a notable portion of these APIs fails to gain market approval despite possessing favorable pharmacological properties.

To tackle this challenge, various approaches have been proposed, such as salt formation [7,8], cyclodextrins [9,10], the formation of pro-liposomes [11,12], and particle size reduction [13]. Besides the aforementioned formulation techniques, there has been significant interest from both academic and industrial researchers in amorphous solid dispersions (ASDs) for their effectiveness in enhancing solubility. Specifically, ASDs can generate drug solution concentrations several times higher than those obtained by the corresponding crystalline form given the absence of long-range order and the presence of high free energy. To date, over 30 products incorporating ASDs have received approval from the US Food and Drug Administration (FDA) and have been introduced into the market [14,15]. Notwithstanding the widespread interest and commercial adoption of ASDs, a notable inherent risk associated with these formulations is the potential crystallization of the amorphous drug given its thermodynamic instability [16]. In this context, the careful choice of suitable matrix(es)/carrier(s) during the design of an ASD is imperative in order to guarantee the physical stability of the system.

An emerging strategy for stabilizing amorphous drugs involves the utilization of mesoporous materials, such as mesoporous silica (MS), as an alternative to polymeric carriers in ASD formulations [17,18,19]. MS, an inert inorganic material with a porous structure ranging from 2 to 50 nm, is available in ordered and non-ordered forms [20,21]. Both exhibit similar surface chemistry, comprising siloxane groups (Si-O-Si) and three types of silanol groups (Si-OH) [22]. The physical stability of ASDs based on MS relies on the absorption of drug molecules into material pores. Specifically, the recrystallization of the amorphous drug is inhibited by interactions that develop between the API and the silanol groups within the pores. These interactions encompass hydrogen bonding, hydrophobic, and/or electrostatic interactions. Furthermore, the physical restriction imposed by the pore structure and the small diameter of the pores—typically below the size of a crystal nucleus—contributes to preventing the recrystallization of drug molecules. However, it is crucial to note that the method used for loading drugs into mesoporous materials can significantly influence the release behavior. Several well-established processes exist for the preparation of MS-based ASDs. Among these, solvent-based methods are more frequently employed owing to their simplicity. These methods either involve suspending the MS in a drug solution or impregnating it through the gradual addition of a concentrated drug solution [23]. Nevertheless, these methodologies present notable challenges, including the extensive use of (organic) solvents, which requires an additional drying step, low yield quantity, and the complexity of the scale-up process. For these reasons, alternative approaches have been suggested, such as temperature-induced solid-phase transformation (e.g., the melt mixing method) [24].

At this point, it is noteworthy that, despite the various in vitro studies that have indicated the capacity of drug-loaded silica to augment the solubility of poorly soluble drugs, only a limited number of studies have showcased enhancements in in vivo absorption [25].

This can be attributed to a limitation in the ability of MS, when employed as the exclusive carrier in ASDs, to hinder the precipitation of supersaturated APIs [18,26]. Consequently, the incorporation of a suitable polymer becomes necessary to sustain the drug supersaturation, a crucial factor for augmenting in vivo oral delivery [27,28,29,30,31]. This approach combines the advantages of the two carriers, achieving synergistic action in improving drug dissolution, bioavailability, and stability [27]. Specifically, in a study by Patel et al. [28], it was demonstrated that ternary ASDs developed with polyvinylpyrrolidone (PVP), and silicon dioxide porous materials exhibited an improved dissolution profile, up to 18 times, compared with the corresponding binary drug–PVP system, while Van Speybroeck et al. [25] demonstrated that the simultaneous administration of hydroxypropyl methylcellulose (HPMC) with itraconazole-loaded mesoporous silicate resulted in a more than 60% increase in absorption compared with the binary system without the addition of the polymeric precipitation inhibitor.

Hence, preceding studies indicate that the development of ternary ASDs utilizing mesoporous silica represents a potentially effective formulation strategy for improving the stability of an amorphous drug within a system and enhancing its dissolution rate. Nevertheless, despite notable progress, significant research efforts are still required to fully harness the capabilities of MSs as suitable matrix/carriers for ASDs.

In this context, the present study attempts to gain further insight into the field by expanding upon the efforts of other research groups in setting the foundations for the in-depth evaluation of MS-based ternary ASDs. Specifically, despite the existence of previously published studies investigating the benefits of ternary MS–polymer ASDs regarding in vitro dissolution performance and physical stability, to the best of our knowledge, our work is the first to specifically focus on the role of the polymer as a constituent of these ternary systems and its interaction with MS, elucidating its impact on the overall performance of ASDs. To accomplish this objective, a direct comparison is undertaken between ternary ASDs containing API–polymer–MS, and the corresponding binary ASDs (either API–MS or API–polymer ASDs) with respect to their physical stability and the rate of drug release from systems. A pharmaceutical substance of BCS Class II, Olanzapine (OLN), was employed as a model drug in this study. OLN, a potent muscarinic M3 receptor antagonist, is widely utilized for its antipsychotic effects, and it ranks among the top 20 prescription drugs [32,33]. Consequently, it is crucial to develop an efficient pharmaceutical dosage form that facilitates an improved pharmacokinetic profile of OLN. To determine the thermal stability profile of the developed systems, thermogravimetric analysis (TGA) was employed, while their thermal properties were assessed through differential scanning calorimetry (DSC). Additionally, to identify changes in the physical state of APIs during the development of ASDs, powder X-ray diffraction (pXRD) was utilized, and the potential development of molecular interactions was assessed via attenuated total reflectance–FTIR (ATR-FTIR) spectroscopy.

## 2. Materials and Methods

### 2.1. Materials

OLN (form II) was purchased from Sigma-Aldrich (Steinheim, Germany). Syloid 244FP silica was obtained from W.R Grace and Co. (Columbia, MD, USA). HPMC (HPMC 15LV, AFFINISOL™, Shin-Etzu, Tokyo, Japan) and copovidone (coPVP) (Kollidon^®^VA64) were kindly given as a gift by Rontis Hellas S.A. (Athens, Greece). All of the other reagents were of analytical or pharmaceutical grade and used as received.

### 2.2. Monomolecular Loading Capacity (MLC) Determination

The determination of MLC was conducted through a methodology previously referred to in other published studies, employing differential scanning calorimetry (DSC) [34,35]. Specifically, physical mixtures (PMs) of OLZ with Syloid 244FP were initially prepared by gentle mixing using a mortar and pestle. The examined ratios of API to Syloid 244FP were 50:50, 60:40, 70:30, 80:20, 90:10, and 100:0 *w*/*w*, with a total quantity of 200 mg. Subsequently, the physical mixtures underwent a DSC heat–cool–heat cycle using a Netzsch DSC 204 F1 Phoenix heat flux instrument (Netzsch, Selb, Germany). The samples were heated at a rate of 20 °C/min to 210 °C, about 10 °C above the melting point (Tm) of OLZ, and held at this temperature for 5 min to ensure the complete fusion of the API into the pores of 244FP. Following this, the samples were quench-cooled to 0 °C, remained there for 3 min, and then reheated to 210 °C. The glass transition temperature (T_g_ midpoint) and the heat capacity change over the glass transition (ΔC_p_) were determined using the NETZSCH Proteus—Thermal Analysis software package (version 5.2.1). A linear fitting of the determined ΔC_p_ as a function of drug fraction (wt%) was performed in order to determine the MLC as the x-intercept (zero ΔCp).

### 2.3. Theoretical Determination of Pore-Filling Capacity (PFC)

In the ternary ASD formulation, the PFC—representing the maximum theoretical amount of the binary system OLN/polymer within the Syloid 244FP pores—was determined by considering the pore volume of Syloid 244FP and the density of the drug and polymers involved, as described in the following equation [35,36,37]:

(1) $$PFC=\frac{V_{244FP\;\mathrm{pore}\;\mathrm{volume}}\times \rho_{(\mathrm{drug}\;\mathrm{and}\;\mathrm{polymer})ASD}}{1+V_{244FP\;\mathrm{pore}\;\mathrm{volume}}\times \rho_{(\mathrm{drug}\;\mathrm{and}\;\mathrm{polymer})ASD}}\times 100$$

where V_244FP pore volume_ was 1.6 cm^3^/g (Syloid 244FP) [22] and ρ_(drug and polymer)ASD_ was determined based on the ratio of OLZ and polymer in the ternary ASD formulation using values of 1.3 g/cm^3^ (amorphous OLZ) and 1.2 g/cm^3^ (for HPMC and coPVP). The PFC includes the drug in the monolayer, as well as the excess drug confined by the pores.

### 2.4. Thermo-Gravimetric Analysis (TGA)

TGA (Shimadzu TGA-50 thermogravimetric analyzer, Tokyo, Japan) was employed in order to evaluate the components’ thermal stability during the ASD preparation process. Briefly, approximately 10.0 mg of the raw materials and the PMs was placed into suitable aluminum sample pans, attached to a sensitive microbalance assembly, and heated from 25 to 300 °C at a rate of 10 °C/min, using nitrogen as purge gas at a flow rate of 50 mL/min. The weight variation of the samples was recorded in relation to temperature, while all experiments were performed in triplicate.

### 2.5. Preparation of the ASDs

For the preparation of binary OLN ASDs (utilizing either of the two examined polymers or Syloid 244 FP) and ternary OLN ASDs (employing the polymeric carriers and Syloid 244 FP), the melt method, a solvent-free technique, was employed. In brief, the requisite quantities of API, polymeric carrier (HPMC or coPVP), and Syloid 244 FP were accurately weighed according to Table 1 (3.0 g of ASD in total). The two specific polymers were chosen in the present study as they are among the most commonly used polymers in the development of binary ASDs. Therefore, it is of interest to investigate their impact when utilized as precipitation inhibitors in MS-based ASDs. Additionally, the thermal properties of both polymers make them excellent carriers for the development of ASDs using melt-based methods [38,39]. As for the choice of the systems’ proportions, it was made considering the results obtained during the determination of the MLC and PFC.

The samples were placed in suitable aluminum pans and melted at 210 °C (approximately 10 °C above the melting point of OLN) on a heating plate for 5 min. Subsequently, the samples were rapidly cooled to −20 °C in a freezer. The binary and ternary ASDs developed were not subjected to any further processing after their cooling, such as grinding. This decision was based on findings from existing studies indicating that pharmaceutical procedures incorporating mechanical stress and pressure may influence the pore structure of porous carriers, with potential repercussions for their efficacy in improving the dissolution rate of poorly soluble drugs [40].

### 2.6. Scanning Electron Microscopy (SEM)

SEM was used to assess the morphological characteristics of the prepared ASDs using a JEOL JSM6390LV scanning electron microscope (JEOL Ltd., Akishima, Japan). All samples were carbon-coated before analysis to provide good conductivity for the electron beam. SEM was performed at an accelerating voltage of 10 kV with a probe current of 45 nA and a counting time of 60 s.

### 2.7. pXRD

The physical state of the prepared ASDs was evaluated via pXRD after grounding them gently using a mortar and pestle. Specifically, pXRD diffractograms of the raw materials and the OLN ASDs were recorded using a D2 PHASER XRD diffractometer (Bruker AXS, Karlsruhe, Germany) with CuKα radiation for crystalline phase identification (λ = 0.15405 nm for Cu Kα). All samples were scanned from 5 to 45° 2θ.

### 2.8. DSC Studies

The thermal properties of the developed ASDs, as well as their corresponding PMs, were characterized through DSC analysis. All DSC measurements were conducted in triplicate using a DSC 204 F1 Phoenix heat flux instrument (Netzsch, Germany). Specifically, the samples (~5.0 mg) were subjected to heating from 25 °C to 210 °C at a rate of 10 °C/min. The melting point of the systems was determined as the onset temperature of the heat flow curve (T_melt,ons_), while the glass transition temperature (Tg) was identified as the inflection point temperature. The enthalpy of fusion (ΔH) was determined by integrating the area under the heat flow curve in all cases Nitrogen flow (50 mL/min) was maintained to ensure a constant thermal environment within the DSC cell. Temperature calibration of the instrument was performed using high-purity indium and tin, while enthalpic calibration utilized indium. Thermograms were analyzed utilizing the Netzsch Proteus–Thermal Analysis software package, version 5.2.1 (Netzsch, Germany). Percent crystallinity was calculated based on the ratio of the melting enthalpy of a sample to that of the pure crystalline form of the API and the mass fraction of the drug [41]

(2) $$\emptyset_{t}=\frac{{∆H}_{f}}{{∆H}_{0}\times {Χ}_{D}}$$

where ΔH_f_ represents the observed melting enthalpy at time t, ΔH_0_ signifies the melting enthalpy of the pure crystalline drug, and X_D_ denotes the proportion of the drug within the sample.

### 2.9. Solvent Shift Method

The efficacy of each polymeric matrix/carrier (i.e., coPVP and HPMC) in preventing the precipitation of OLN in a phosphate buffer solution (PBS), pH 6.8, was investigated by employing the solvent shift method. This method was adapted from previously published studies [42].

Initially, an incremental introduction of approximately 5 mL of a 10% *w*/*v* OLN solution in methanol into a dissolution medium consisting of 500 mL of the chosen buffer was conducted, with or without the incorporation of a polymer and Syloid 244 FP. In cases where the buffer included a polymer, the polymer concentration was set at 10 mg/mL. The concentration of Syloid 244 FP in PBS 6.8 was exactly the same in cases where it was introduced into the system, either alone or in combination with a polymer.

At designated time intervals (5, 10, 15, 30, 45, 60, 90, 120, 180, and 240 min), about 3 mL aliquots were withdrawn and subsequently subjected to centrifugation at 17,586× *g* for 3 min. The resulting supernatant underwent filtration through a 0.45 μm hydrophilic membrane (Sartorius, Göttingen, Germany, Model Minisart RC 25) and was diluted with methanol before analysis using a UV/VIS spectrometer (Shimadzu, Kyoto, Japan) at a wavelength of 270 nm. All tests were performed in triplicate.

### 2.10. Molecular Interactions

Possible molecular interactions evolving within the ASDs were systematically assessed via ATR-FTIR spectroscopy. For the ATR-FTIR analysis, the spectra of the initial raw materials, the physical mixtures (PMs), and the ASDs were prepared according to Table 1 and recorded utilizing a Shimadzu IR-Prestige-21-FT-IR infrared spectrometer (Tokyo, Japan) coupled with a horizontal Golden Gate MKII single-reflection ATR system (Specac, Kent, UK) after appropriate background subtraction. For every spectrum, 64 successive scans were received in the 700–4000 cm^−1^ region (at a resolution of 4 cm^−1^), and from the average of these measurements, the final spectrum was obtained.

### 2.11. OLN Equilibrium Solubility in Dissolution Medium

The equilibrium solubility studies of crystalline OLN were conducted in triplicate. This involved introducing an excess amount of OLN into 100 mL of the dissolution medium, specifically, PBS with a pH of 6.8. The resultant solution underwent stirring at 100 rpm and was maintained at a temperature of 37 °C for 24 h. Following this, the sample was filtered through 0.45 μm polyvinylidene fluoride (PVDF) filters and subsequently analyzed spectrophotometrically at 270 nm using a UV/VIS spectrometer (Shimadzu, Kyoto, Japan). All tests were performed in triplicate.

### 2.12. Non-Sink Condition Dissolution Studies

Supersaturated dissolution studies of the prepared ternary ASDs, along with the neat crystalline OLN, were conducted on an apparatus II dissolution tester (PT-DT7 Pharma Test AG, Hainburg, Germany). The experiments were conducted in 200 mL of PBS at pH 6.8, maintaining a constant temperature of 37 °C and a paddle rotation rate of 100 rpm. Non-sink conditions were maintained during all dissolution tests in order to build up the supersaturation, as it commonly occurs under finite volume conditions in the GI tract. The extent of departure from sink conditions was quantified using a dimensionless sink index (SI), calculated through the following equation:

(3) $$SI=\frac{C_{s}\times V}{Dose}$$

where C_s_ represents the solubility of the crystalline API, V denotes the volume of the dissolution medium under examination, and Dose signifies the total quantity of the drug in the sample. For this study, a uniform reference SI value of 0.5 was established across all instances. OLN’s concentration was determined spectrophotometrically at 270 nm. All studies were performed in triplicate.

### 2.13. Physical Stability under Storage

The prepared binary and ternary ASDs (~2 g) were placed in open vials within desiccators at elevated humidity levels (60 ± 5% RH) at room temperature. Subsequently, analysis was conducted after intervals of three and eight months to evaluate any potential modifications in the physical state of OLN utilizing pXRD and DSC using the methodologies and conditions described previously.

## 3. Results

### 3.1. Investigation of PFC and MLC

As indicated in the introduction section, the primary mechanisms for stabilizing amorphous APIs within MS involve two key aspects: (1) the development of non-covalent interactions between drug molecules and the MS surface and (2) the confinement of the drug within the pores of the MS [43]. Consequently, the influence of the MS’s surface area and pore volume on drug-loading capacities becomes evident. In greater detail, the substantial MS surface area introduces additional surface free energy, and it has been proposed that the absorption of the drug in its amorphous state is thermodynamically advantageous [44]. When all binding sites on the MS surface are occupied by drug molecules and an excess amount of drug is present, it can no longer be in direct contact with the MS surface. In this scenario, the drug begins to fill up the pores, and this surplus amorphous drug may be stabilized by physical restraint from crystallization. Consequently, the loading capacity of a drug in MS can be categorized into two classes: (a) the drug in direct contact with the MS surface, forming a drug monolayer (referred to as the MLC), and (b) any excess drug filling up the pores (referred to as the PFC) [37]. Any further addition of the drug beyond these limits results in overloading, with the drug present outside of the pores behaving as a neat amorphous drug.

However, a notable drawback is the inconsistent reporting of drug loading capacity, even for the same drug–MS system. This inconsistency may arise from the utilization of different loading techniques [34]. For instance, Limnell et al. observed varying loading capacities of indomethacin in Syloid 244 FP, specifically, 11.6% *w*/*w* and 28.9% *w*/*w*, when employing two distinct solvent-based loading techniques [45]. In this context, Hempel et al. [34] proposed a DSC-based method to experimentally determine the MLC. This approach involves deliberately overloading the MS with the drug during melting and quenching (using drug loadings of 50–90 wt%). Subsequently, the heat capacity change (ΔC_p_) over the glass transition temperature (T_g_) of the excess drug, specifically, the portion beyond the monolayer, is determined upon reheating. Given that the monolayer does not contribute to the T_g_ signal in the DSC, the MLC is determined by extrapolating the ΔC_p_ values for various drug loading levels to zero (the x-intercept).

**Figure 1:**
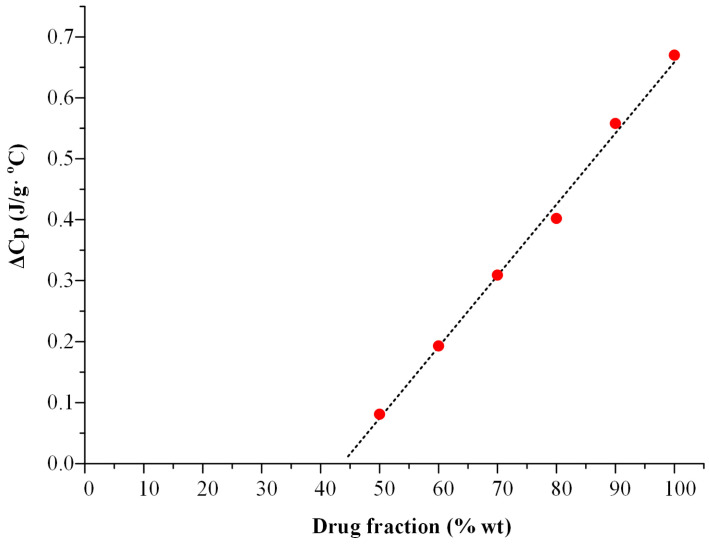
Heat capacity (ΔC_p_) points (red dots) plotted as a function of OLN fraction for Syloid 244 FP after the heat-cool–heat cycle in the DSC. The data are extrapolated to zero ΔC_p_ through linear extrapolation (solid dashed line, r^2^ = 0.9963).

The ΔC_p_ extrapolation for the OLN-Syloid 244 FP system is depicted in Figure 1. For this system, the MLC (the x-intercept of the extrapolated function) was found to be 43.8 wt%. Regarding the ternary systems, the PFC was calculated using Equation (1) and determined to be 66.7%.

Considering these findings and acknowledging that the monolayer is in a more thermodynamically favorable state, it is theoretically expected that systems with OLN content lower than or up to 43.8% will demonstrate superior thermodynamic stability. Conversely, an excess of amorphous drug within the pores (i.e., OLN content higher than the MLC but below the PFC) is stabilized by the small pore diameter, which physically impedes the crystallization of the drug within the pore. As outlined above, this pertains to one of the two stabilization mechanisms employed for the amorphous drug within the MS-based system. This mechanism relies on the spatial confinement of the drug within the pores of MS [20,36,46]. The specific spatial constraint is applied to the clusters of molecules prior to reaching the critical nucleation size. As a result, the nucleation and growth of crystals are impeded, ensuring that the system maintains an inherently non-crystalline state. Nevertheless, this portion of the drug lacks thermodynamic stability since its crystalline form represents the lowest energy state [37].

To practically investigate these theoretical boundaries, two distinct API concentrations were chosen and examined within OLN-244 FP ASDs. One concentration was below the MLC, precisely in the OLN-244 FP 3:7 system, while the other was higher, specifically in the OLN-244 FP 1:1 system.

### 3.2. Thermal Stability via TGA

As mentioned, the melt method was employed for the development of OLN ASDs with Syloid 244 FP. This decision was guided by published research indicating superior efficiency and higher filling percentages achieved through the melting method for loading APIs onto MS compared with immersion methods [47]. The diminished efficiency observed in the latter methods is predominantly attributable to the competitive interaction between the solvent and the drug during the pore-filling process [48,49].

**Figure 2:**
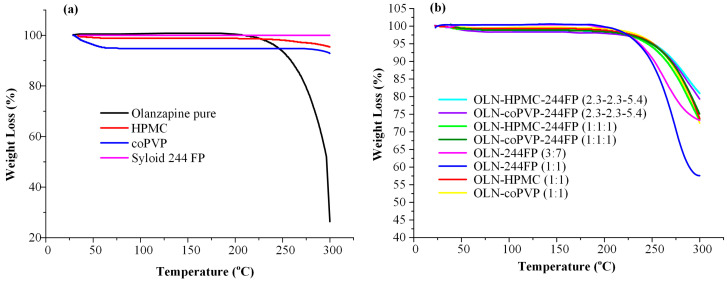
TGA thermograms of all raw materials (**a**) and binary or ternary OLN PMs at various weight ratios (**b**).

However, any loading method based on melting necessitates the thermal stability of the drug and, generally, the individual components of the systems during processing at elevated temperatures. In this context, the thermal stability profile of the raw materials was examined through TGA. As indicated by the results presented in Figure 2a, OLN remains stable up to ~210 °C, while the thermal degradation of HPMC initiates at approximately 260 °C. Syloid 244 FP exhibits thermal stability up to 300 °C, while coPVP shows only a slight weight loss (∼4.0% *w*/*w*) at temperatures below 100 °C, indicative of residual moisture water loss. Regarding the PMs of the binary and ternary OLN systems that were investigated, the results (Figure 2b) indicated that the binary OLN-Syloid 244 FP systems exhibited a similar thermal degradation path to pure OLN for both weight ratios studied.

On the other hand, the remaining examined PMs, in which a polymer was incorporated (either alone or in combination with Syloid 244 FP), showed a slightly improved thermal stability profile. These results demonstrate that the temperature selected during the melt process (i.e., 210 °C) does not lead to any thermal degradation of the API or matrixes/carriers.

### 3.3. Morphology Analysis

**Figure 3:**
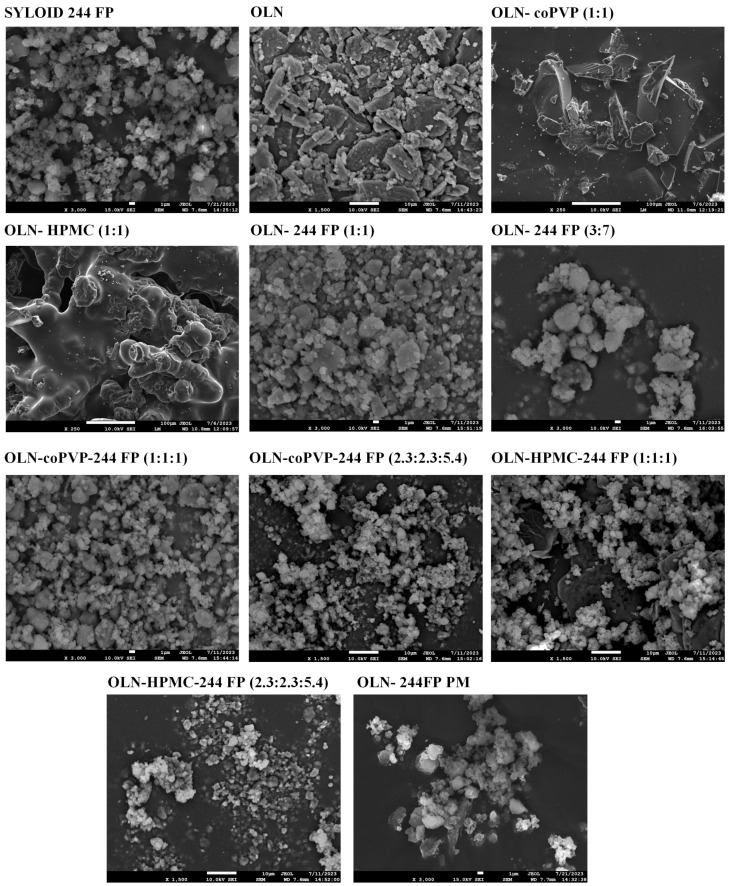
SEM micrographs of pure Syloid 244 FP, crystalline OLN, and OLN ASDs.

The morphology of the prepared OLN ASDs was evaluated via SEM, and the images collected are presented in Figure 3. Syloid 244 FP appears to consist of very fine, irregularly shaped, and loosely aggregated particles, while pure OLN can be observed as small to large irregularly sized and shaped crystals with a tendency to self-agglomerate. These findings are consistent with references to previously published studies [22,50]. The binary OLN ASDs with coPVP or HPMC exhibit a uniformly compact shape without the appearance of porosity. OLN ASDs containing Syloid 244 FP, whether in binary or ternary formulations, display a comparable morphology, appearing as aggregated particles.

### 3.4. pXRD

**Figure 4:**
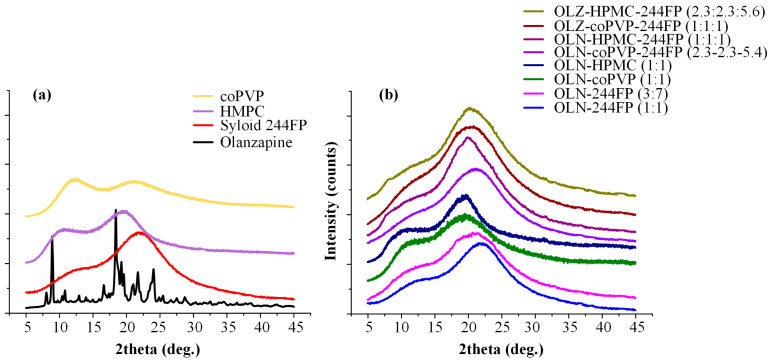
Powder X-ray diffractograms for the raw materials (**a**) and prepared binary and ternary OLN ASDs (**b**).

Subsequently, the successful formation of ASDs in the developed binary and ternary OLN systems was evaluated through pXRD analysis. In Figure 4a, the pXRD diffractograms of all pure components, as well as the binary and ternary OLN systems at zero time from their preparation, are presented. The pXRD diffractogram of OLN indicated that the API exhibits high crystallinity, as evidenced by the several characteristic 2θ diffraction peaks observed at 10.92°, 12.99°, 14.03°, and 19.38°. According to published studies, these peaks can be attributed to the Form ΙI structure of the API [51]. Regarding Syloid 244 FP and both studied polymers (i.e., coPVP and HPMC), the obtained pXRD patterns revealed their amorphous state, as evidenced by the presence of two broad halos in the pXRD patterns of the polymers and a single broad amorphous halo for Syloid 244 FP. These results are in absolute agreement with previously published studies, where both Syloid 244 FP [52] and the polymers under investigation were characterized as amorphous [53,54]. Concerning the binary and ternary OLN systems, successful ASD formation was confirmed in all cases, as no characteristic reflection peaks of the API were observed in the initial patterns obtained at zero time (Figure 4b). The absence of Bragg peaks in a pXRD diffraction pattern implies the absence of crystalline material [55].

### 3.5. DSC Studies

**Figure 5:**
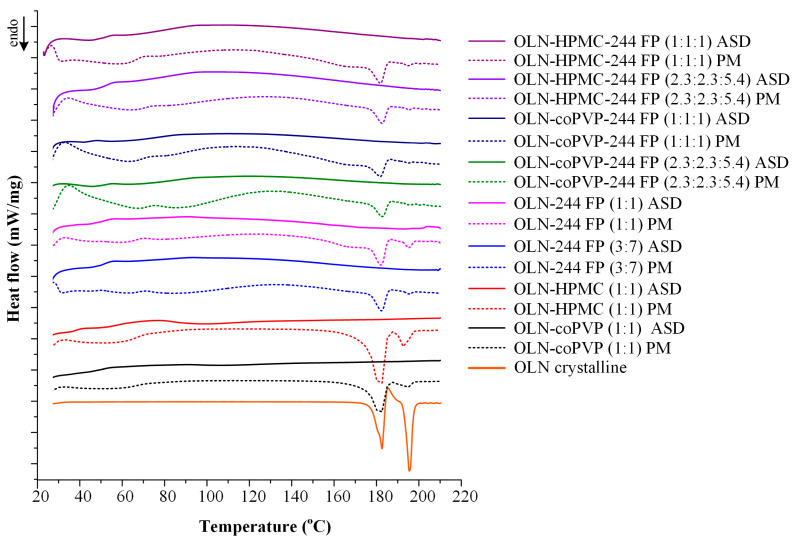
DSC thermograms of crystalline pure OLN, the developed ASDs, and their corresponding PMs.

In addition to pXRD analysis, the physical state of the systems was investigated through DSC. Specifically, the objective of the DSC studies was to confirm the absence of even minimal residual crystallinity in the developed samples (<10%), which could not be detected by pXRD analysis. For this purpose, the magnitude of melting endotherms was utilized to assess the extent of API crystallization in the systems. In Table 2, the thermal properties of the PMs are presented, while the DSC thermograms of the ASDs, as well as their corresponding PMs in comparison with the thermogram of pure OLN, are presented in Figure 5. Crystalline OLN exhibits a characteristic melting peak at 179.5 °C with a ΔH_f_ of 85.65 kJ/mol, corresponding to the melting peak of polymorph II in the API [56], followed by a recrystallization exotherm at T_cryst_ 185.6 °C and a subsequent melting endotherm (form I crystals) at T_melt,ons_ 193.6 °C. As evident from the figure, the absence of the OLN melting peak is apparent in all studied systems, indicating the absence of crystallinity, even in minimal amounts (<10%). These findings are fully consistent with the pXRD results presented above and confirm the successful formation of ASDs in all cases.

Finally, Table 2 provides a comprehensive overview of the thermal properties of the PMs studied via DSC. It is evident that both the polymers and Syloid 244 FP exhibit plasticizing effects on the API, as evidenced by the shifting of the OLN’s melting peak to lower temperatures in both the binary and ternary systems. Additionally, as expected, an increase in API content corresponds to an increase in ΔH_f_ due to the higher crystallinity of the system.

### 3.6. Anti-Precipitant Screening Using the Solvent Shift Method

**Figure 6:**
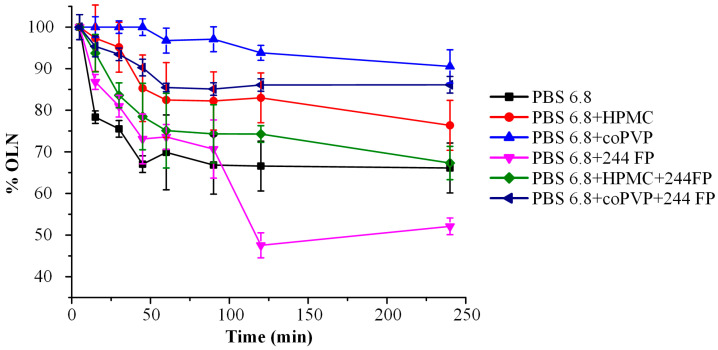
OLN concentration profile during the evaluation of matrixes/carriers based on the solvent.

The solvent shift method is recognized as the optimal approach for identifying a suitable anti-precipitant for poorly water-soluble compounds. This method is acknowledged for its simplicity and efficiency, eliminating the requirement for volatile organic solvents such as alcohol and acetone [57]. The concentration changes in OLN over time, with or without the tested matrix/carriers, are illustrated in Figure 5. The results indicate that coPVP is the most effective precipitation inhibitor for OLN, maintaining OLN concentration at higher levels compared with other investigated systems. Notably, the outcomes presented in Figure 6 reveal that Syloid 244 FP acts as a precipitation enhancer for the API. This is evidenced by the lower OLN concentrations observed in the dissolution medium compared with the neat API alone, signifying an inferior performance. The reduction in the degree of supersaturation of OLN in the presence of Syloid 244 FP could be attributed to various potential phenomena, such as a decrease in the saturation solubility of OLN in the dissolution medium in the presence of Syloid 244 FP or even a minor alteration in the viscosity of the solution, which could affect the molecular mobility of the API and, hence, its crystallization tendency [58].

In the case where the precipitation of OLN was examined in the ternary systems (i.e., OLN-Syloid 244 FP in combination with coPVP or HPMC), according to our findings, a higher concentration of OLN was observed in the case of Syloid 244 FP-coPVP. This finding reaffirms that, in comparison with HPMC, coPVP serves as a more appropriate inhibitor in preventing the precipitation of OLN. This phenomenon could be attributed to various mechanisms, such as the increased hydrophilicity of coPVP compared with HPMC [59] or the possible development of stronger molecular interactions between the API and coPVP, leading to the reduced molecular mobility of the drug and, thus, a decreased tendency for recrystallization [60].

At this point, it is imperative to emphasize that, during the conduction of the anti-precipitant screening studies utilizing the solvent shift method across the examined systems, the concentrations of the polymers, Syloid 244 FP, and their ratios with the API were deliberately kept identical. This measure was taken to enable a direct comparison of the carriers’ effectiveness in preventing the precipitation of the supersaturated API. The selection of these parameters was based on previously published studies [42,61].

### 3.7. In Vitro Release Studies

In order for an ASD system to be considered stable, it is essential not only to exhibit physical stability during storage but also to possess the ability to achieve and maintain the supersaturated state of the drug during dissolution. However, the dissolution of an ASD is a complex process involving multiple stages and can be influenced by various factors.

The typical dissolution profile of an ASD initially shows a rapid increase in drug concentration followed by a subsequent reduction due to the recrystallization of the pharmaceutical substance and its precipitation. This phenomenon is known as the “spring and parachute” effect, initially proposed by Guzman et al. [62]. The additional free energy encountered in the amorphous state acts like a spring, driving the concentration of the active substance to a level higher than the solubility of its crystalline form, resulting in a supersaturated solution. The supersaturated state observed is followed by a decrease in drug concentration in the dissolution medium due to precipitation. However, the matrix/carrier acts as a “parachute,” preventing a rapid decrease in the concentration of the pharmaceutical substance. Therefore, an appropriate polymer for preparing ASDs should behave as a “strong parachute” and aid in maintaining the supersaturated state by minimizing the precipitation of the active substance during its stay in the gastrointestinal tract.

In this context, several research groups have evaluated whether conducting dissolution studies under *non-sink* conditions is a promising method for gaining a deeper understanding of drug supersaturation kinetics and potential in vivo precipitation. In an attempt to ascertain the degree of departure from sink conditions in dissolution experiments involving supersaturated ASDs, Sun et al. introduced the dimensionless index, SI [63]. Specifically, a high SI value represents a dissolution system close to satisfying the sink condition requirement, whereas a lower SI value (e.g., SI ≪ 3) indicates that the dissolution study is conducted under non-sink conditions. As demonstrated by various studies, the rate and extent of supersaturation significantly impact the resulting dissolution kinetic profiles of supersaturated dosage forms. In this study, the dissolution studies utilized a selected SI value of 0.5. According to the results, the equilibrium solubility of OLN at pH 6.8 was 0.4 mg/mL.

**Figure 7:**
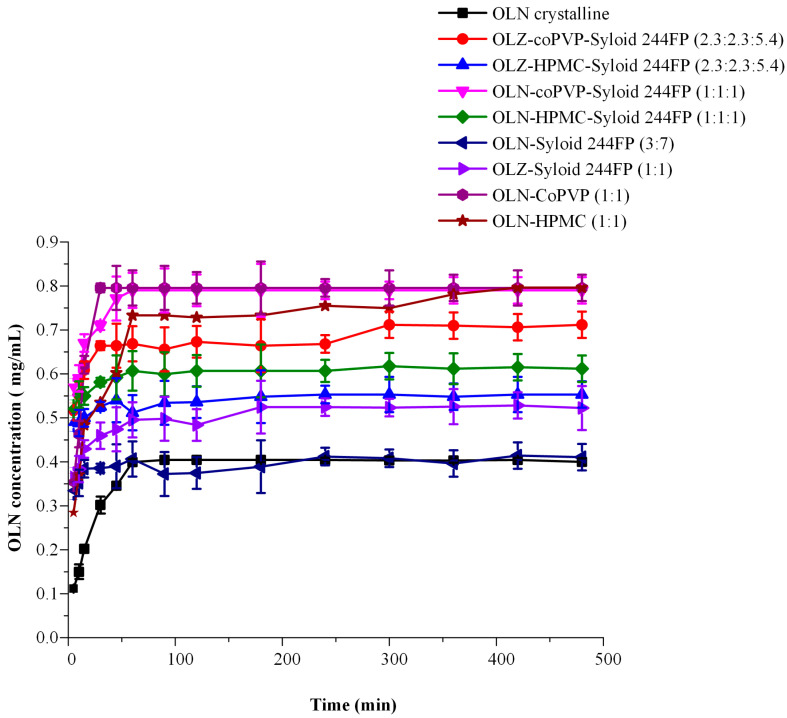
In vitro dissolution profiles of amorphous OLN, binary OLN ASDs, and ternary OLN ASDs at different weight ratios. The red dashed line represents the saturation solubility of OLN.

In Figure 7, the in vitro dissolution profiles of binary and ternary OLN ASDs are presented in comparison with the corresponding profile of pure crystalline OLN.

According to the obtained results, it appears that, despite the successful development of ASDs in all cases and regardless of the carrier and its content, a significant differentiation can be observed in the in vitro dissolution profile of the systems.

In the case of binary OLN-Syloid 244 FP systems, the results indicated that the concentration of OLN released from the ASD system OLN-244 FP, with a ratio of 3:7, reached a plateau equal to the saturation solubility of crystalline OLN. This observation implies that Syloid 244 FP was unable to impede the recrystallization of the API into its thermodynamically stable crystalline form. Contrastingly, in the case of the OLN-244 FP (1:1) system, the observed plateau exceeded the equilibrium solubility of the crystalline form, signifying the establishment of OLN supersaturation. However, a significantly lower degree of OLN supersaturation was noted compared with the binary or ternary systems containing either coPVP or HPMC. This observation can be directly associated with the results obtained using the solvent shift method, where Syloid 244 FP when examined solely without the presence of a polymer, appeared incapable of inhibiting the precipitation of the API. In addition, this is in full agreement with findings reported in previously published studies where the incorporation of a polymeric carrier into an MS-based ASD leads to an improved in vitro dissolution profile [35,40]. Furthermore, it is evident that the proportion of API to MS impacts the ultimate drug concentration obtained during dissolution investigations, demonstrating an elevated concentration at the highest OLN:Syloid 244 FP ratio (i.e., 1:1). This observation concurs with findings presented in the work of Lai et al. [64], wherein the influence of drug loading on the in vitro release performance of ibuprofen from mesoporous carriers was explored.

Regarding the ternary OLN ASDs, a higher degree of supersaturation appears to be achieved in the systems where coPVP is utilized as a precipitation inhibitor. Specifically, the OLN-coPVP-244 FP system (1:1:1) exhibits a similar in vitro dissolution profile to the binary ASD OLN-coPVP system, with complete API release observed within the initial hour. The observation of increased drug release from ASDs formulated with coPVP compared with those with HPMC is consistent with findings from prior studies that investigated the influence of these polymers on inhibiting drug precipitation and the dissolution behavior of ASDs, yielding comparable outcomes [59,65]. The superior ability of coPVP, compared with HPMC, to generate and maintain supersaturated drug solutions was attributed to the differences observed in the hydrophilicity of the two polymers. Specifically, according to the wetting kinetics, coPVP exhibits superior wetting properties and a predominantly hydrophilic nature. In contrast, HPMC exhibits a combination of hydrophilic and hydrophobic characteristics [59].

The superiority of the coPVP-based ternary ASDs was further validated through the calculation of the area under the curve (AUC(0→t)) for all dissolution profiles. The results, as summarized in Table 3, exhibited a more than twofold increase in the AUC(0→t) compared with the crystalline OLN for the ternary ASDs developed using coPVP as the polymeric carrier. It is noteworthy that, at this point specifically, the OLN-coPVP-244 FP (1:1:1) system released 100% of the entrapped API. Similarly, the ternary ASDs developed with HPMC also demonstrated an increase in OLN release compared with the pure drug, albeit lower than the enhancement achieved with the coPVP in vitro release profile improvement. In conclusion, the results confirm that the OLN-244 FP (3:7) system exhibited a very marginal (borderline) increase in AUC(0→t) (i.e., 1.01 higher than crystalline OLN).

### 3.8. Physical Stability

**Figure 8:**
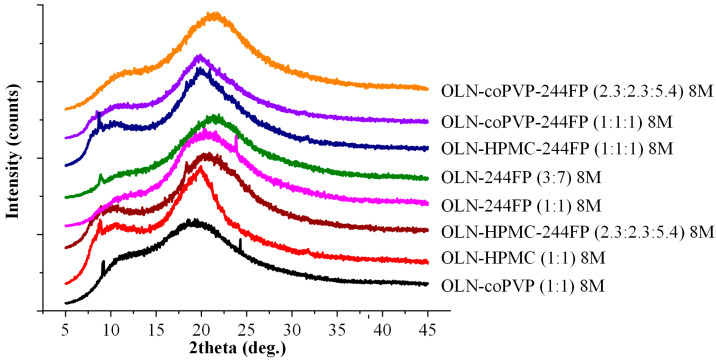
pXRD diffractograms of binary and ternary OLN ASDs after 8 months of storage (8 M).

The pXRD diffractograms of the OLN ASDs after storage for 8 months under elevated humidity conditions (25 °C/60 ± 5% RH) are presented in Figure 8. According to the obtained results, the binary OLN ASDs developed with Syloid 244 FP as the sole carrier exhibited signs of API recrystallization, indicating reduced physical stability. Interestingly, the crystallization of OLN occurred in both the examined OLN-244 FP ASD systems, regardless of whether the drug content was below or above the theoretical limit of the MLC.

Similarly, reduced physical stability can also be observed in ASDs developed with HPMC, both in the binary and ternary systems. Conversely, the ternary OLN ASDs developed utilizing coPVP and Syloid 244 FP as carriers/matrices, regardless of the component ratios, remained stable after 8 months of storage, showing no indications of API recrystallization. At this point, it should be noted that, when coPVP was examined alone, i.e., in the absence of Syloid 244 FP, it did not demonstrate significant efficacy as an inhibitor of API recrystallization. A corresponding pXRD diffractogram revealed specific characteristic peaks of OLN, indicating the reduced stability of the system during storage. This indication is consistent with the existing indications, as extensively discussed in the introduction, which suggests that the incorporation of a suitable polymer into MS-based ASDs functions synergistically in sustaining drug supersaturation and enhancing the physical stability of ASDs. An illustrative example is provided in the study by Hanada et al., where the physical stability of ternary amorphous solid dispersions using HPMC and Syloid as carriers was investigated. The study indicated that HPMC alone as a carrier is not sufficient for maintaining a highly mixed ASD at a high drug load without the assistance of Syloid [66].

**Figure 9:**
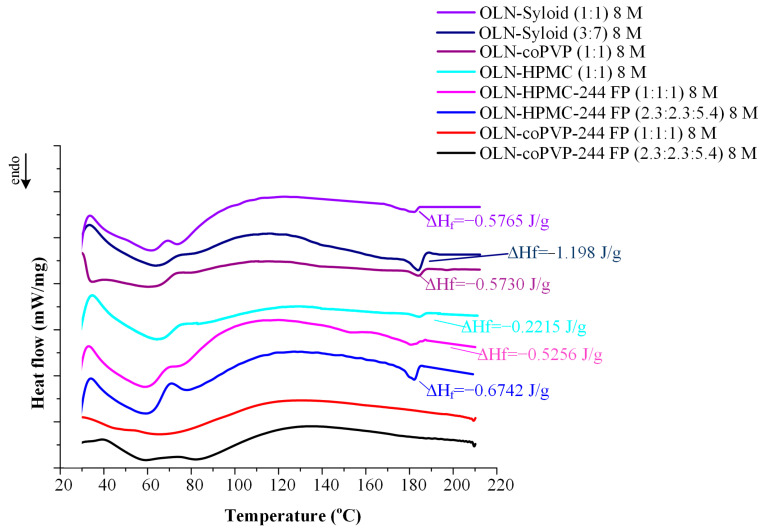
DSC thermograms of binary and ternary OLN ASDs after 8 months of storage (8 M).

Figure 9 depicts DSC thermograms of the ASDs following storage. As evident from the graph, the only systems not exhibiting the OLN melting peak are the ternary ASDs: OLN-coPVP-244FP. This finding confirms the results of the pXRD analysis, where these ternary systems were the only ones to remain amorphous after an 8-month storage period. The enhanced physical stability of these systems could be attributed to the presence of stronger molecular interactions between the components, resulting in the inhibition of the crystallization of the API. However, it should be noted that the incorporation of HPMC into the system resulted in lower ΔH_f_ values compared with the binary OLN-244FP ASDs. Nonetheless, this carrier was not sufficient for fully inhibiting the crystallization of OLN, as observed with the coPVP-Sylloid 244 FP carrier combination. Furthermore, it should be emphasized at this point that the broad peaks observed in a temperature range of 40–100 °C can be attributed to the water absorbed by the samples during their storage at elevated humidity levels (60 ± 5% RH).

Hence, based on the obtained results, it is evident that the combination of coPVP with Syloid 244 FP exhibits a synergistic effect on the physical stability of this amorphous API. These findings can also be linked to the results of in vitro dissolution studies, where ternary OLN-coPVP-244 FP ASDs exhibited the highest degree of supersaturation compared with the other ternary ASDs, indicating that these systems are capable of inhibiting an API’s precipitation. However, the in vitro dissolution performance of ASDs is a more challenging process because of the presence of the dissolution medium, which can significantly influence the molecular interactions between the carrier and the API [67].

Nevertheless, it is conceivable that, in systems exhibiting OLN crystallization during the storage period, a diminished level of supersaturation can be anticipated in comparison with time zero [68].

### 3.9. ATR-FTIR

Considering that the development of molecular interactions between the drug and the matrices/carriers in the ASDs stands as a fundamental stabilization mechanism for this amorphous API, their presence in the developed OLN ASDs was investigated through ATR-FTIR spectroscopy in an attempt to elucidate the superior performance observed in the ternary OLN-coPVP-244 FP systems.

**Figure 10:**
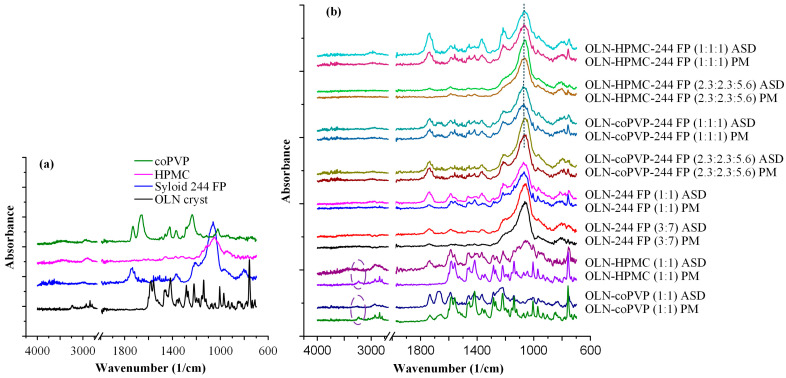
ATR-FTIR spectra of (**a**) the raw materials, (**b**) the binary and ternary drug physical mixtures (PM), and the ASDs of OLN.

First, the spectra of the pure materials are illustrated in Figure 10a. For pure crystalline OLZ, the ATR-FTIR spectrum presented a characteristic peak of N-H stretching at 3239 cm^−1^, C-H stretching at 2931 cm^−1^, a C=C aromatic ring symmetric stretch at 1583–1556 cm^−1^, a C=C aromatic ring asymmetric stretch at 1468–1446 cm^−1^, C-H bending at 1411 cm^−1^, and C-N stretching at 1289 cm^−1^ and 1223 cm^−1^. coPVP displayed characteristic peaks at 3447 cm^−1^ corresponding to absorbed water, 1450–1480 cm^−1^ corresponding to C-C binding, and 1658 and 1728 cm^−1^ corresponding to the ν(C=O) of 1-vinyl-2-pyrrolidone and vinyl acetate, respectively. Prominent ATR-FTIR peaks identified in HPMC were at 2922 cm^−1^, ascribed to C-H stretching; 3420 cm^−1^, indicative of O-H stretching; and 1058 cm^−1^, attributed to C-O stretching. Finally, the ATR-FTIR spectrum of pure Syloid 244 FP exhibited broad bands at 1060 cm^−1^ and 802 cm^−1^, signifying silanol-bending (Si-OH). These data are in complete agreement with previously published results pertaining to ATR-FTIR analyses of the specific raw materials [22,35,54,69,70].

As for the ATR-FTIR spectra of the PMs and the ASDs of OLN, these are depicted in Figure 10b. Regarding the ATR-FTIR spectra of the binary OLN ASDs formulated with the polymeric matrix/carriers (i.e., coPVP and HPMC), a notable disappearance of the OLN peak at 3293 cm^−1^ can be observed in comparison with the ATR-FTIR spectra of the corresponding PMs. Given that this characteristic peak can be attributed to the -NH group of the API, which serves as a hydrogen bond donor for the drug substance, it can be inferred that hydrogen bonding interactions form between the drug and the studied polymers [71]. In the case of the ATR-FTIR spectra of the OLN ASDs containing Syloid 244 FP, a shift in the silanol peak can be observed, indicating the formation of molecular interactions among the components of the systems [72]. Looking more closely at the obtained results, in the case of the OLN-coPVP-244 FP systems, specifically with ratios of 2.3:2.3:5.4 and 1:1:1, an FTIR peak shift can be observed from 1062 to 1058 and 1056, respectively. Similarly, in the OLN-HPMC-244 FP systems with ratios of 2.3:2.3:5.4 and 1:1:1, a peak shift is noted from 1068 to 1066 cm^−1^ and from 1062 cm^−1^ to 1060 cm^−1^, respectively. Therefore, the more pronounced peak shift observed in the case of the ternary ASDs developed with the polymeric carrier coPVP, especially at the 1:1:1 ratio, can be considered to indicate the development of stronger molecular interactions in these systems compared with those developed with HPMC [73]. It can be inferred that these are hydrogen bonds given that the primary functional groups on the silica surface are predominantly silanol groups, which can function as both hydrogen bond donors and acceptors, thereby establishing hydrogen bonding as the primary interaction mechanism [74].

Finally, a hypothesis can be proposed stating that, in this system, the development of stronger molecular interactions may potentially explain its superior performance in in vitro dissolution studies and its remarkable physical stability during storage.

## 4. Conclusions

The use of mesoporous materials has been proposed as a viable approach for stabilizing amorphous drugs. Nevertheless, these carriers do not ensure the prevention of the precipitation of the supersaturated API and, hence, they are not considered to guarantee enhanced in vivo absorption. To address this limitation, the development of ASDs using a combination of a polymer and a mesoporous material as a carrier is suggested. In this context, the present study explores the development of OLN ASDs with Syloid 244 FP, integrating coPVP or HPMC into the system. Initially, it is crucial to emphasize that, in all investigated systems, the physical state studies conducted via both DSC and pXRD confirmed the successful formation of ASDs. Notably, there was no discernible crystallinity observed in any of the systems immediately after their preparation.

The impact of introducing polymers as the tertiary constituents in OLN-Syloid 244 FP systems was examined concerning the efficacy of ASDs in both in vitro dissolution studies and their physical stability. Of the two polymers under investigation, coPVP appears to be an optimal polymeric carrier during the development of these systems. Specifically, the addition of coPVP to OLN ASDs containing Syloid 244 FP leads to a twofold increase in the degree of drug supersaturation compared with pure crystalline API. These systems also exhibited nearly double the API release compared with that observed from the corresponding binary OLN-Syloid 244 FP ASDs.

This underscores the efficacy of the polymer in preserving the supersaturated state of OLN. Additionally, the ternary OLN-coPVP-Syloid 244 FP ASDs demonstrated significantly improved physical stability in comparison with the binary systems involving OLN with either coPVP or Syloid 244 FP as carriers, indicating a synergistic influence between the two carriers in enhancing the stability of the amorphous API. Specifically, the ternary OLN-coPVP-244FP ASDs were identified as the only systems that did not exhibit crystallinity after storage for 8 months under elevated humidity conditions. The presence of strong intermolecular H-bonds between the components (identified and evaluated by ATR-FTIR spectroscopy) can be provided as an explanation for this synergistic effect. Hence, it can be inferred that developing ASDs using a combination of a polymer and a mesoporous carrier may prove to be a highly promising technique for enhancing the solubility of poorly soluble drugs, maintaining their supersaturated state, and increasing the physical stability of these systems.

## Figures and Tables

**Table 1:** Composition of the prepared ASDs.

Formulation ID	OLN% *w*/*w*	coPVP% *w*/*w*	HPMC% *w*/*w*	Syloid 244 FP% *w*/*w*
OLN-coPVP (1:1)	50.00	50.00	-	-
OLN-HPMC (1:1)	50.00	-	50.00	-
OLN-244 FP (3:7)	30.00	-	-	70.00
OLN-244 FP (1:1)	50.00	-	-	50.00
OLN-coPVP-244 FP (1:1:1)	33.33	33.33	-	33.33
OLN-coPVP-244 FP (2.3:2.3:5.4)	23.00	23.00	-	54.00
OLN-HPMC-244 FP (1:1:1)	33.33	-	33.33	33.33
OLN-HPMC-244 FP (2.3:2.3:5.4)	23.00	-	23.00	54.00

**Table 2:** Thermal properties of PMs.

Formulation ID	PM		
	T_m,ons_ (°C)	T_m,peak_ (°C)	ΔH_f_ (J/g)
Olanzapine	179.5 (form II)	182.5 (form II)	85.65 (form II)
	193.6 (form I)	195.8 (form I)	85.58 (form I)
OLN-coPVP (1:1)	170.1 (form II)	181.5 (form II)	49.92 (form II)
	190.9 (form I)	192.9 (form I)	6.30 (form I)
OLN-HPMC (1:1)	176.7 (form II)	182.2 (form II)	47.86 (form II)
	190.4 (form I)	193.7 (form I)	6.82 (form I)
OLN-244 FP (3:7)	178.7 (form II)	182,5 (form II)	9.18 (form II)
	193.8 (form I)	195.3 (form I)	0.53 (form I)
OLN-244 FP (1:1)	177.5 (form II)	182,8 (form II)	14.59 (form II)
	194.2 (form I)	195.3 (form I)	2.30 (form I)
OLN-coPVP-244 FP (1:1:1)	178.6 (form II)	182.4 (form II)	6.25 (form II)
	193.9 (form I)	195.5 (form I)	0.62 (form I)
OLN-coPVP-244 FP (2.3:2.3:5.4)	177.4 (form II)	182.4 (form II)	5.05 (form II)
	194.9 (form I)	195.9 (form I)	0.55 (form I)
OLN-HPMC-244 FP (1:1:1)	179.7 (form II)	182.9(form II)	9.00 (form II)
	193.0 (form I)	194.9 (form I)	0.72 (form I)
OLN-HPMC-244 FP (2.3:2.3:5.4)	177.9 (form II)	182,6 (form II)	8.24 (form II)
	193.9 (form I)	195.1 (form I)	0.71 (form I)

**Table 3:** Summary of in vitro dissolution-estimated AUC (0→t) for the crystalline OLN and the binary and ternary OLN ASDs, along with the estimated mean AUC(0→t) ratio (i.e., AUC(0→t)[Sample]/AUC(0→t)[OLN, cryst]).

Formulation ID	ID AUC_(0→t)_ [μg/mL min ×10^2^] (Mean ± SD)	AUC_(0→t)_ Ratio (Mean)
OLN crystalline	1.85	1.00
OLN-coPVP (1:1)	3.73	2.02
OLN-HPMC (1:1)	3.48	1.88
OLN-244 FP (3:7)	1.88	1.01
OLN-244 FP (1:1)	2.41	1.30
OLN-coPVP-244 FP (1:1:1)	3.71	2.01
OLN-coPVP-244 FP (2.3:2.3:5.4)	3.24	1.75
OLN-HPMC-244 FP (1:1:1)	2.87	1.55
OLN-HPMC-244 FP (2.3:2.3:5.4)	2.58	1.39

## Data Availability

Data available on request due to privacy.
